# Insights on the upper mantle beneath the Eastern Alps

**DOI:** 10.1016/j.epsl.2014.06.051

**Published:** 2014-10-01

**Authors:** Irene Bianchi, Meghan S. Miller, Götz Bokelmann

**Affiliations:** a Institut für Meteorologie und Geophysik, Universität Wien, Althanstraße 14 (UZA II), 1090 Vienna, Austria; b Department of Earth Sciences, University of Southern California, Los Angeles, CA 90089-0740, USA

**Keywords:** upper mantle, Eastern Alps, low velocity zone, lithosphere–asthenosphere boundary, P-receiver functions, S-receiver functions

## Abstract

Analyses of Ps and Sp receiver functions from datasets collected by permanent and temporary seismic stations, image a seismic discontinuity, due to a negative velocity contrast across the entire Eastern Alps. The receiver functions show the presence of the discontinuity within the upper mantle with a resolution of tens of kilometers laterally. It is deeper (100–130 km) below the central portion of the Eastern Alps, and shallower (70–80 km) towards the Pannonian Basin and in the Central Alps. Comparison with previous studies renders it likely that the observed discontinuity coincides with the lithosphere–asthenosphere boundary (LAB) east of 15°E longitude, while it could be associated with a low velocity zone west of 15°E.

## Highlights

• Coherent results from P- and S-RF analysis, for the lithosphere under the E. Alps.
• RFs show a discontinuity in the upper mantle due to a negative velocity contrast.
• The variation in lithosphere is attributed to the tectonic history of the E. Alps.

## 1 Introduction

The Alps are the result of long term convergence between the Eurasian and African plates, which began around 120 Ma ago (DeMets et al., 1994). Subduction initiated ∼80 Ma ago and plate collision started 35 Ma ago (Handy et al., 2010, and references therein) followed by uplift of the Alpine orogenic belt after 23 Ma (e.g. Schmid et al., 2004, Castellarin and Cantelli, 2000). As expected, such a long series of tectonic processes have led to the formation of a highly complex and heterogeneous structure of the crust (Hirn et al., 1980, Pfiffner, 1990, Ye et al., 1995, Bleibinhaus and Transalp Working Group, 2001, TRANSALP Working Group, 2001, TRANSALP Working Group et al., 2002) and the upper mantle (Hirn et al., 1984, Panza et al., 1980, Pfiffner et al., 1988, Kissling, 1993, Lippitsch et al., 2003, Koulakov et al., 2009, Bokelmann et al., 2013).

Tomographic models of the upper mantle beneath the convergent zone (Wortel and Spakman, 2000, Piromallo and Morelli, 2003, Giacomuzzi et al., 2011) have determined the current position of ancient suture zones by imaging high-velocity anomaly bodies running parallel to the Alpine chain axis that extend into the mantle transition zone. Regional tomographic models (Lippitsch et al., 2003, Mitterbauer et al., 2011) show that the positive velocity anomalies (ascribed as the Alpine slab) are interrupted along the Alpine chain, testifying the presence of fragmented subduction. Seismic models based on P-wave residuals (Babuška et al., 1998), MT and electromagnetic studies (cf. Jones et al., 2010, Korja, 2007), as well as geothermal (mostly steady-state) models (Artemieva et al., 2006, and references therein) describe an anomalously thin (60–80 km) lithosphere below the Pannonian Basin. Previous works show that the lithosphere is thickened beneath the Bohemian Massif to 120–140 km (Babuška and Plomerová, 2001, Heuer et al., 2007; Geissler et al., 2010, Geissler et al., 2012; Plomerová et al., 2012), which is a large stable body of crystalline rock representing the eastern part of the European Variscan Orogen. The whole mantle in this area has been imaged by P receiver function studies of Kummerow et al. (2004) and Hetényi et al. (2009). However in Kummerow et al. (2004) the occurrence of the Moho multiples obscures possible conversions due to structures within the upper mantle, while Hetényi et al. (2009) primarily explore the mantle transition zone and irregular lateral variation of the 660 discontinuity due to accumulation of cold and denser material, described as subducting slabs actively impinging on the lower mantle. Receiver functions computed along longitude ∼12°E show the thickening of the European crust towards south until the central part of the Eastern Alps (Kummerow et al., 2004).

The surface expression of the arcuate Alpine belt can be divided into two distinct blocks; the arcuate western Alps, and the Eastern Alps, which extends towards the east to the Carpathians. There are many open questions regarding the eastern part of the Alpine belt. Is it linked at depth to the Carpathian arc or to the Dinaridic arc? Does a Pannonian plate exist and is there a triple junction between Europe, Adria, and Pannonia (Brückl et al., 2010)?

Is it possible to distinguish the effects of the indentation and of the lateral extrusion (e.g. Ratschbacher et al., 1991a)? In this paper we investigate the structure of the shallow portion of the upper mantle, down to 150 km, beneath the central and eastern Alps, to contribute additional information that may help addressing some of these outstanding questions, in particular by looking at the occurrence of a decrease in seismic velocities. Sharp velocity reductions in the upper mantle have been suggested to be due to the boundary that separates the lithosphere from the asthenosphere (LAB) (Barrell, 1914, Eaton et al., 2009) or to a mid-lithospheric discontinuity (MLD) globally located in a depth interval below 100 ± 20 km (Thybo and Perchuć, 1997, Thybo, 2006, Abt et al., 2010, Lekic and Romanowicz, 2011). A distinct velocity drop was globally identified by P receiver functions between 70 ± 4 km in oceanic environments, and down to 95 ± 4 km beneath Precambrian shields and platforms (Rychert and Shearer, 2009); possible explanation for these observations has been proposed by Karato (2012) that considering the amount of water content, the geothermal gradient and the grain boundary sliding, identifies a decrease in seismic velocities in the depth range between 70 (oceans) and 150 (continents) km. In this work we present P and S receiver function results that detect a shallow negative velocity contrast in the mantle below the Eastern Alps and its depth variations that occur over length scales of several tens of kilometers. A negative velocity contrast implies the occurrence of a seismic discontinuity below which the S-velocity decreases. The results for the two different methodologies are strikingly similar.

## 2 Data and methods

### 2.1 P receiver functions

**Fig. 1:**
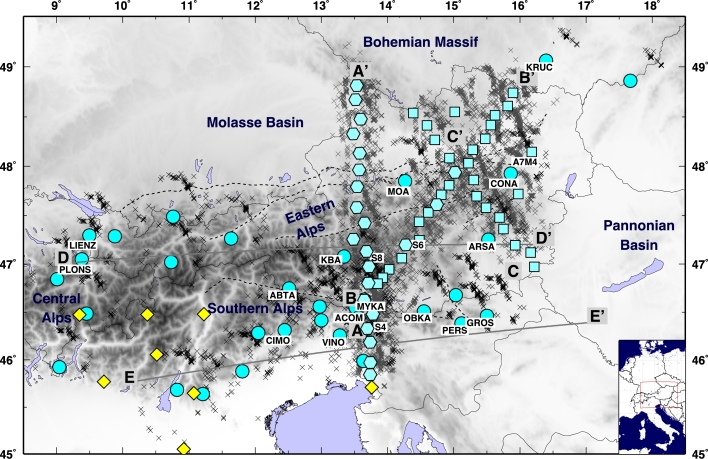
Map of the Eastern Alps, including seismic station locations. Light blue circles show broadband permanent stations from different national networks, hexagons for broadband temporary stations, and squares for short period temporary stations. Yellow diamonds show stations used in Miller and Piana Agostinetti (2012). Gray crosses are the piercing points at 100 km depth for PRF. Black crosses are piercing points at 100 km for SRF. The inset shows the study area location in Central Europe. (For interpretation of the references to color in this figure legend, the reader is referred to the web version of this article.)

More than 8000 waveforms, from 536 teleseismic events with Mw ≥ 5.5 occurred at epicentral distances between 30° and 100°, were used to compute the P receiver functions. Waveforms from these events were recorded at 56 three-components stations (Fig. 1), 53 belonging to the ALPASS temporary network (Mitterbauer et al., 2011) which were deployed between July 2005 and April 2006, and 3 stations from the Carpathian Basin Project (CBP) temporary network that were deployed from May 2006 to June 2007 (Dando et al., 2011).

**Fig. 2:**
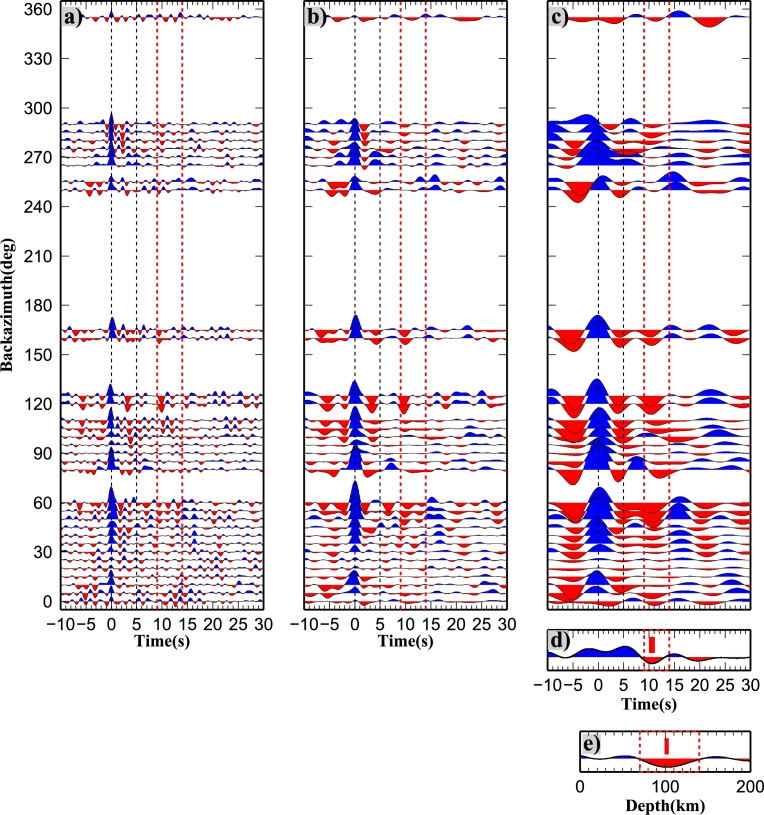
Example of PRFs calculated for “spot” 8 along profile AA′. (a), (b), (c) Stacks of single PRFs displayed according to backazimuth for different cut-off frequencies (in (a) at 1 Hz, in (b) at 0.5 Hz and in (c) at 0.2 Hz), in RTZ reference system; red dotted lines highlight the negative pulses contributing to the construction of the negative pulse highlighted in (d) and (e). (d) Stack of PRFs rotated in LQT. (e) Stack of PRFs migrated at 100 km depth. In (d) and (e) the red vertical bar shows the location of the minimum used to infer the discontinuity depth. (For interpretation of the references to color in this figure legend, the reader is referred to the web version of this article.)

The P receiver function (PRF) method isolates the effect of mode conversions generated at velocity discontinuities at depth beneath a seismic station. The technique has been employed primarily for determining the depth of the crust–mantle boundary (Moho) but has also been widely used for imaging other discontinuities such as the LAB or the mantle transition zone (e.g. Rychert and Shearer, 2009, Hetényi et al., 2009). PRFs are the result of P-to-S (Ps) waves generated by the conversion of the incoming teleseismic P-wave into an S-wave by the passage through a seismic interface at depth (Langston, 1979, Ammon, 1991). The presence of several velocity jumps at depth causes the presence of several Ps converted phases together with their multiples (such as PpPs, PsPs + PpSs phases); consequently a more complicated structure results into a more phase-populated receiver function. In this study, P receiver functions have been calculated in the RTZ reference system with a frequency domain algorithm using multitaper correlation estimates (Park and Levin, 2000) with a frequency cut off of 0.2 Hz in order to image the deep lithospheric structure. This method provides an estimate of PRF uncertainty in the frequency domain, using the pre-event noise spectrum for frequency-dependent damping. The multitaper spectrum estimates are leakage resistant, so low-amplitude portions of the P-wave spectrum can contribute usefully to the PRF estimate. This enables PRFs from different seismic events to be combined in a weighted-average PRF estimation according to the inverse of their variance. The weighted average PRFs are obtained by binning events in 10° bins for both epicentral distance and from backazimuth (Fig. 2). For each PRF we calculated the mean and standard deviation (*σ*) as in Abt et al. (2010). We consider the well-resolved portions of the receiver functions to be those still significant at positive amplitudes below the rf−σ and negative amplitudes above the rf+σ (see supplementary online material). An example of the events distribution for PRF is shown in Fig. S1.

To enhance the continuity of structures in the study area, we used a Common Conversion Point stacking technique (Dueker and Sheehan, 1998, Wilson et al., 2004) following the procedure described in Bianchi et al. (2010), along the three main profiles (AA′, BB′ and CC′) crossing the Alps in various directions (Fig. 1). For each profile, we divide the area within 50 km of the profile into rectangular boxes of 20 km width that overlap by 50% (i.e. each area shares 50% of its surface with the adjacent areas). For each rectangular area, we select the ensemble for PRFs whose surface projections of conversion points at a fixed depth are located inside the rectangular area (each bin collects between 107 and 942 receiver functions). We associate the PRFs with the center of the rectangular area (termed as “spot”) and then migrate the PRFs using the IASP91 model (Kennett and Engdahl, 1991) to 100 km depth, which approximates the 1D velocity structure of the continental lithosphere.

About half of the ALPASS stations (in profiles BB′ and CC′) were equipped with short-period instruments (see Fig. 1). RF calculated with data from short-period instruments have been previously published (e.g. Jones and Phinney, 1998, Nakamichi et al., 2002, Knapmeyer-Endrun et al., 2013) and show that when the data are stacked into a beam, do retain coherent conversions and suppress incoherent energy. In Fig. S2 we show the frequency content of both broadband and short-period instruments. At frequencies lower than 0.2 Hz, amplitudes from the short-period instruments are lower with respect to the broadband, and for this reason we stacked for a larger number of events when stations equipped with a short-period sensor are included in the profile (i.e. profiles BB′ and CC′); due to the smaller number of RF gathered in the North-western part of profile CC′, computed PRFs display an error larger than the signal, for this reason they are not included in this analysis. We stack multiple events to ensure that we sample a variety of ambient noise conditions, leading to enhanced signal by cancellation of the noise; we exploit the close station spacing of the array to enhance confidence in the signal by stacking events recorded at adjacent stations.

**Fig. 3:**
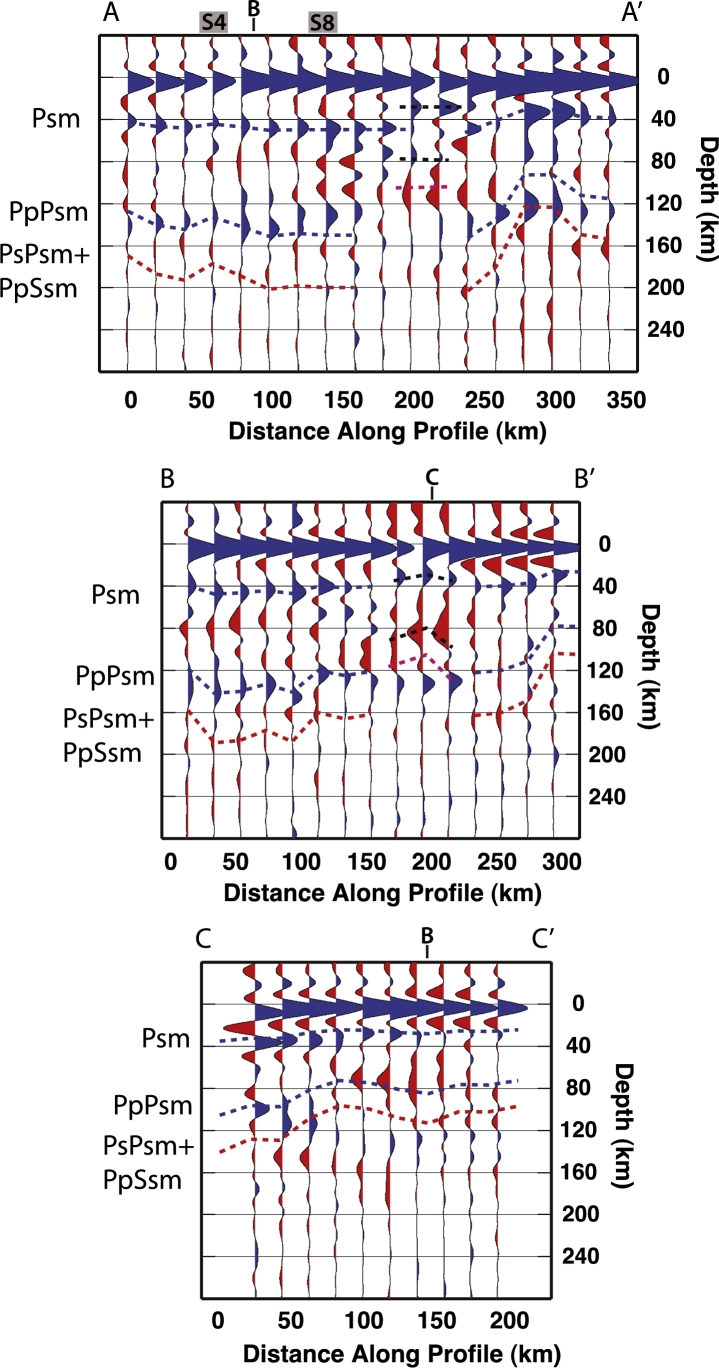
PRF common-conversion-point (CCP) profiles migrated at 40 km depth along profiles AA′, BB′ and CC′ (see Fig. 1 for locations). “Spot” 4 and 8, as well as profiles crossings are marked. PRF are computed with a frequency cut off of 0.5 Hz. The most prominent converted phase (Psm) (occurring between 30 and 50 km depth on the three profiles) has been recognized as the Moho. Blue dashed line highlights the Psm along the profiles. Predicted arrival times of the Moho multiples are marked by a further blue dashed line (PpPsm phase) and by a red dashed line (for the negative PsPs + PpSs phase). Black dashed lines mark the arrival of a converted phase at a crustal interface, its multiples are marked by a further black line, and by a purple dashed line for the negative amplitude multiple. (For interpretation of the references to color in this figure legend, the reader is referred to the web version of this article.)

In order to validate the quality of our data, we show the three profiles AA′, BB′ and CC′ migrated at 40 km depth (in Fig. 3). The most prominent Ps phase (occurring between 30 and 50 km depth on the three profiles) has been recognized as the Moho; at 200–240 km within profile AA′ and at 150–200 km within BB′, the phase marked by a black dashed line, does not correspond to the Moho estimates by Brückl et al. (2007) and Grad et al. (2009), this phase might therefore be due to an intracrustal conversion. The blue dashed line highlights the Moho phase along the profiles. Predicted arrival times for the Moho multiples are displayed in the figure and labeled as PpPs (for the earlier positive multiple) and PsPs + PpSs (for the later negative multiple).

An example of the PRFs at the common-conversion point “spot” 8 along the profile AA′ is shown in Fig. 2. Fig. 2a, b, c shows radial PRF bins for different cut-off frequencies, along a backazimuthal sweep. The Moho phase arrives at 5 s, is quite clear at 1 Hz and the energy associated with it decreases with the decreasing cut-off frequency value, then becomes mostly undetectable with cut-off frequencies below 0.2 Hz. The lower frequency content of data (up to 0.2 Hz) is considered better for imaging at greater depths (as in e.g. Chen et al., 2006). A negative phase is observed at 10 to 12 s. The stacked PRF (Fig. 2d) is rotated into the LQT coordinate system to enhance the converted phases, and shows the minimum of the negative phase at 11 s delay time. The stacked and migrated image (Fig. 2e) shows that the migration process emphasizes the negative phase. Synthetic examples of the PRFs obtained for 1, 0.5 and 0.2 Hz cut-off frequencies for two velocity models, one including a S-velocity decrease at 80 km depth, and another displaying no velocity jumps at 80 km depth are shown in Fig. S3. Multiples from shallower layers might have an arrival time similar to the conversion from the S-velocity decrease, but this example shows a 15% larger amplitude negative phase due to the velocity decrease at depth. By stacking for a large number of PRF and migrating at 100 km depth, multiple phases due to crustal structures are destructively interfering, and coherent phases are constructively interfering.

### 2.2 S receiver functions

More than 2000 records of events with magnitude (Mw) greater than 6.0 occurred at an epicentral distance between 55° and 85°, were used in our S receiver function analysis (Fig. S2). The seismic stations (Fig. 1) are a combination of broadband stations from the different national seismic networks: 12 stations of the Austrian seismological permanent network (operated by ZAMG, Zentralanstalt für Meteorologie und Geodynamik) with records between January 2009 and June 2011; 10 stations from the north Italian network NI maintained by Istituto Nazionale di Oceanografia e di Geofisica Sperimentale (OGS) with records from December 2006 to December 2011; 2 Slovenian stations (SNRS network) with records from December 2006 to December 2012; 5 stations of the Swiss Digital Network (SDSNet) with records since April 2006 to December 2012; 2 stations belonging to the Institute of Geophysics of the Academy of Sciences of the Czech Republic (IG-CAS) with records from January 2009 to December 2012. All data have been downloaded through ORFEUS (Observatories and Research Facilities for European Seismology, www.orfeus-eu.org).

S-receiver function (SRF) analysis has become as an effective method for mapping the lithospheric structure and thickness both globally and in regions of active tectonics (i.e. Sodoudi et al., 2006, Sodoudi et al., 2011; Li et al., 2007, Abt et al., 2010, Levander and Miller, 2012, Miller and Piana Agostinetti, 2012). Similar to PRFs, SRFs isolate the S-to-P (Sp) waves generated by the conversion of the incoming teleseismic S-wave into a P-wave by the passage through a seismic interface at depth. Yet, SRFs are lower in frequency in comparison to PRFs and the paths of the incoming S-wave are considerably longer and more oblique than those of the incoming P-wave. However, the advantage of using SRFs is the lack of contamination from multiples, which are common in PRFs, as converted P phases appear as precursors to the main S phase (Yuan et al., 2006); multiple P waves may arrive in front of the S wave at the same time as S-to-P conversions (Bock, 1994) but because of the different slowness they are canceled during the migration and stacking process. Although SRFs cannot consistently resolve intra-crustal structure due to their relatively low frequency nature, they are well suited for determining depths to the crust–mantle boundary and deeper structures such as the lithosphere–asthenosphere boundary.

We follow the methodology described in detail in Levander and Miller (2012) and Miller and Piana Agostinetti (2012). S waves on each of the three components of the seismogram were visually inspected for clear S-wave arrivals in the appropriate time window, then the time series were transformed from Z, N, and E to P, SV, and SH components. The SRFs were produced by deconvolving the SV component from the P component in the frequency domain following Langston (1979). Then the amplitudes were reversed, so that the positive amplitudes indicate an impedance increase, such as the Moho, allowing for easier comparison to P receiver functions. The receiver functions were depth-converted using the 1D velocity model IASP91 (Kennett and Engdahl, 1991), to be consistent with the PRFs, but also because it effectively represents the continental lithosphere. Although the Alpine velocity structure is complex, the use of a 1D model is appropriate for resolving the level of structure imaged in SRFs, as shown in synthetic tests (Miller and Eaton, 2010, Zhai and Levander, 2011, Levander and Miller, 2012). Then in the final processing step, the receiver functions were corrected using a cross-correlation method for residual statics introduced by near-surface irregularities, and stacked as a sum at each station. For each SRF, as for the PRF, we calculated the mean and standard deviation (*σ*). Due to the smaller distance range, in comparison to PRFs (55° to 80°), we used teleseismic events that primarily come from the northeast (see examples in Fig. S1).

**Fig. 4:**
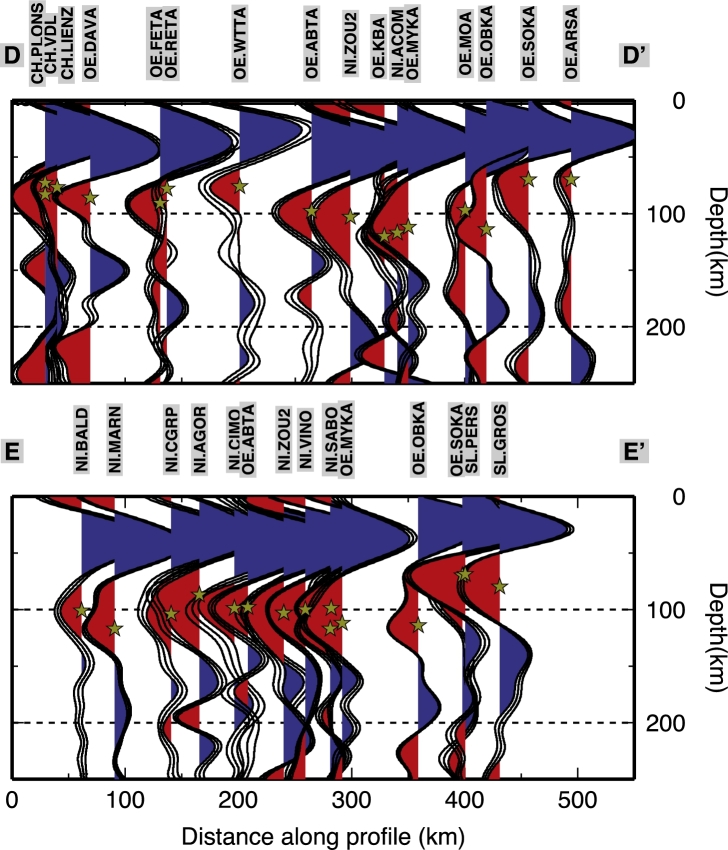
SRF stacks for the permanent stations along profiles DD′ and EE′ (see Fig. 1, Fig. 9 for locations). Stars indicate the interpreted discontinuity depth at each station. SRF are filtered with an upper frequency of 0.2 Hz. In each RF, the middle black line is the mean receiver function from the bootstrap, and the thin black lines on either side of the mean are the bootstrap ± 2 standard deviations, details in Appendix A. Stations name and network are indicated on top of each SRF. (For interpretation of the references to color in this figure, the reader is referred to the web version of this article.)

**Fig. 5:**
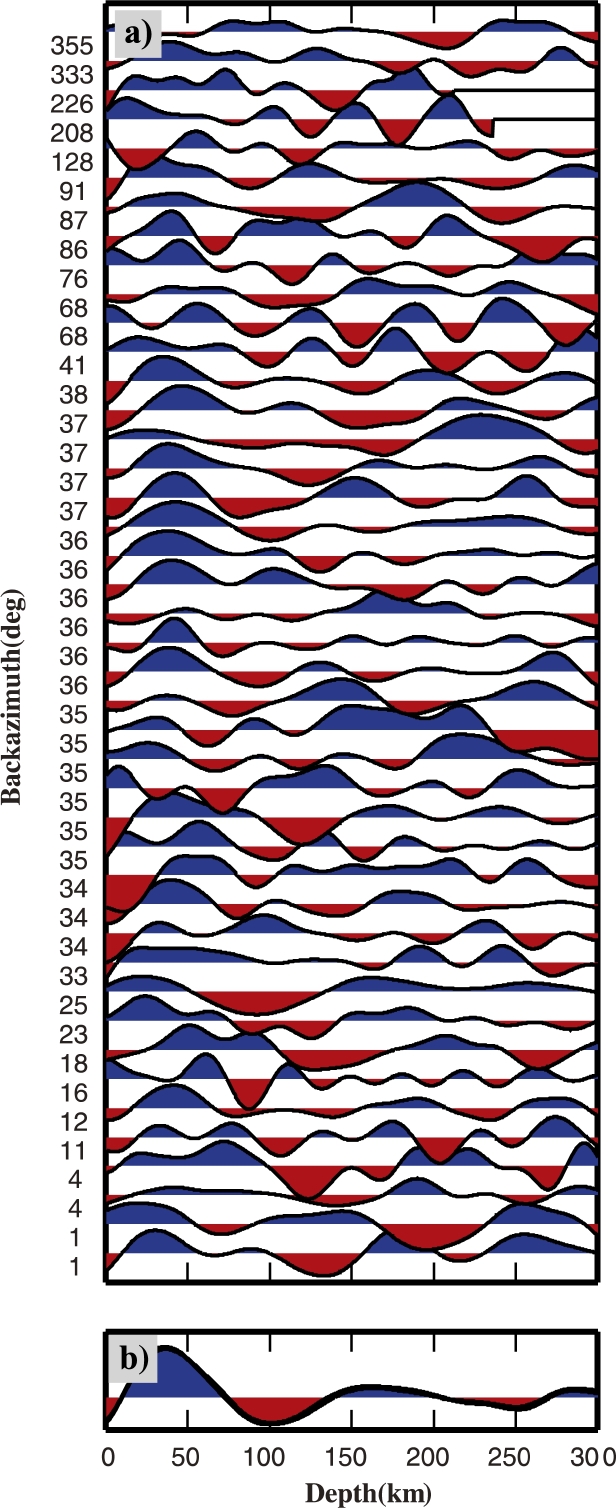
S-receiver functions calculated for station VINO (see Fig. 1 for location), filtered with an upper frequency of 0.2 Hz. (a) SRFs displayed according to the origin backazimuth. (b) Stack of SRFs.

**Fig. 6:**
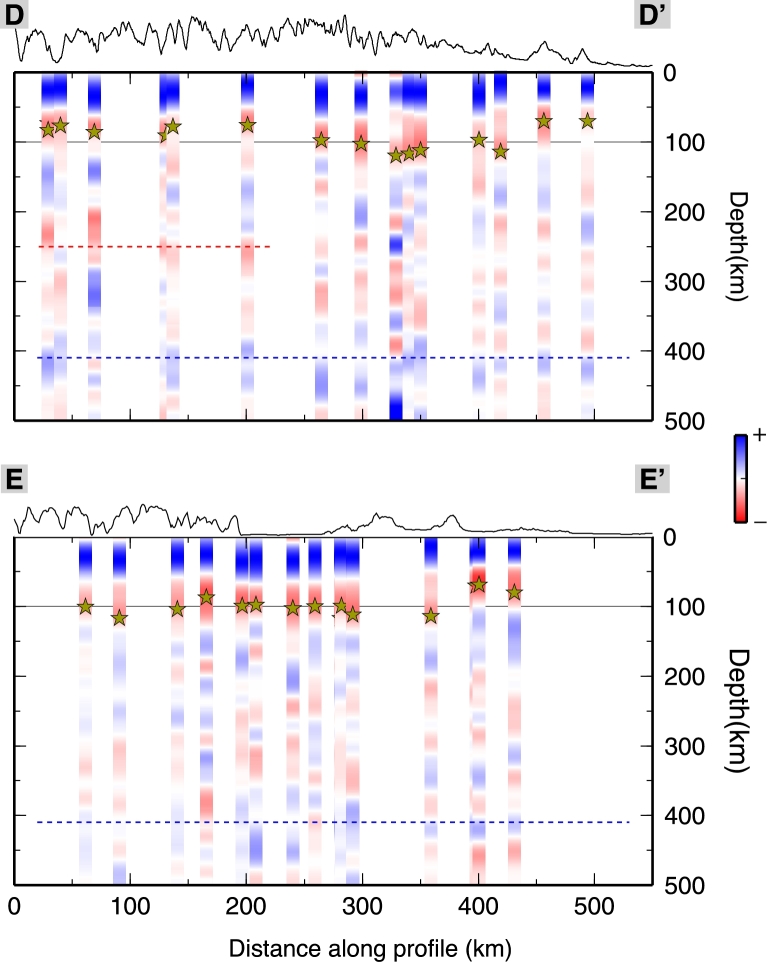
Amplitudes for SRF computed at the permanent stations and displayed along profiles DD′ and EE′ down to 500 km depth (see Fig. 1, Fig. 9 for locations), SRF are filtered with an upper frequency of 0.2 Hz. Stars indicate the interpreted discontinuity depth at each station. The red dashed line highlights an S velocity decrease at larger depth. Blue dashed line marks the 410 km discontinuity. (For interpretation of the references to color in this figure legend, the reader is referred to the web version of this article.)

The paths of the S waves from the estimated locations of depth conversion to the surface, where they are recorded, are longer with respect to the analogous P-wave paths (as shown in Fig. 1 for the piercing points at depth of the P and S waves). Due to the more oblique incidence angle, the conversions occur farther away from the surface location of the station. Nevertheless, the construction of sub-groups of SRF stacks generates wiggles with much larger standard deviation values, resulting from a signal from where is hard to decipher a clear negative phase. For this reason, and because of the backazimuthal coverage for the SRFs that are not well sampled, we prefer to stack all the collected SRFs at each station, and determine an average discontinuity depth for the negative velocity contrast. The backazimuthal subdivisions would require a larger amount of data that are not available at the moment. Fig. 4 shows the depth converted SRFs computed for each station along two profiles. All SRFs show an initial blue (positive) pulse due to the S-to-P conversion through the Moho interface. All are characterized afterwards by the presence of a red (negative) pulse witnessing the occurrence of a strong velocity reduction. An example of SRF computed for data recorded at the station VINO are shown in Fig. 5. The inferred negative discontinuity depth estimate is 100 km. For the range of uncertainties refer to Table S1 and Appendix A. The SRFs are shown for depths to 500 km for completeness in Fig. 6. A positive polarity is recognized along the two profiles coinciding with the 410 km discontinuity. Besides the negative pulse described in Fig. 4, we pick a deeper negative phase at 250 km depth in the western part of profile DD′ at 0–200 km within profile. Further negative phases have not been interpreted since they show smaller amplitudes and little consistency along profiles.

## 3 Results

**Fig. 7:**
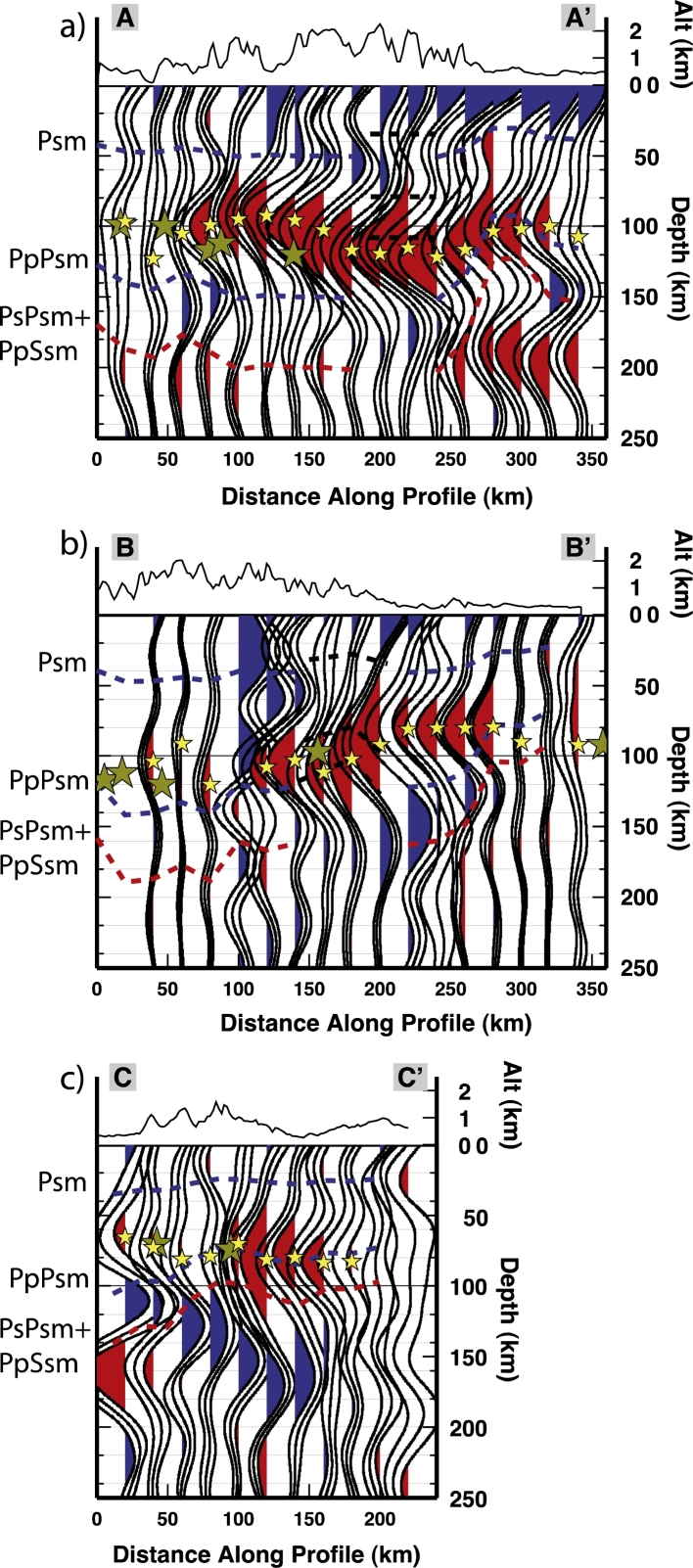
PRF common-conversion-point (CCP) profiles migrated at 100 km depth along profiles AA′, BB′ and CC′, PRF are computed with a frequency cut off of 0.2 Hz (see Fig. 1, Fig. 9 for locations). The discontinuity contribution is highlighted thanks to the depth migration: green stars for depth from the SRFs at adjacent stations. Yellow stars indicate the interpreted discontinuity depths from the PRFs. Blue and red dashed lines mark the arrival times of the Moho phase (Psm) and its multiples (PpPs and PsPs + PpSs), black dashed lines mark the arrival times of the intracrustal phase and its multiples, as in Fig. 3. (For interpretation of the references to color in this figure legend, the reader is referred to the web version of this article.)

Using both SRF and PRF analyses, we detect a negative impedance contrast generated by a discontinuity in the upper mantle. Results from the PRFs are analyzed along three CCP profiles migrated at 100 km depth (Fig. 7). The closely located TRANSALP RF profile, at ∼12°E longitude (Kummerow et al., 2004) suggests the Moho multiples obliterate the shallow part of the mantle, not allowing the detection of the LAB or low velocities layers. We display arrival times of the Moho phase and its multiples in Fig. 7. The marked phases and multiples predicted arrival times have been extracted from profiles AA′, BB′ and CC′ migrated at 40 km depth (Fig. 3). The negative phase that we interpret in this study is marked in Fig. 7 with yellow stars and arrives with a delay time in between the Psm and its multiples. At 200–240 km within AA′, 150–200 km within BB′ and 100–120 km within CC′, the negative multiples (PsPs + PpSs) merge with the converted phase, broadening the phase due to the velocity decrease. The discontinuity retrieved depths from single stations SRF analysis are strongly coincident with depths estimated from the PRFs although some scattering in the results is observed at the crossing between AA′ and BB′. This may be due to the location of the piercing points of the events used to compute the SRF. Indeed the conversions do not happen beneath the stations but at some distance away, therefore the rays might sample different structures. In the Southern Alps, stations MYKA, ACOM, VINO detect the discontinuity at a depth of ∼105–110 km which are comparable to the depths estimated by the PRFs for the southern part of the AA′ transect (see Fig. 7a). The discontinuity depth estimates based on the SRFs at the stations MYKA, ACOM, KBA, MOA and KRUC also correspond to the estimated depths from PRFs along profile BB′ (Fig. 7b). Strong agreement among the results is observed for the easternmost area, where the discontinuity depth values for station ARSA and CONA are also extremely consistent to the shallow depth estimates from the PRFs obtained for the south-eastern part of the CC′ profile (Fig. 7c). In the SRF profiles shown in Fig. 4 there is a deepening of the discontinuity signal in the central part of profile DD′ (ABTA to OBKA), and a nearly flat (∼100 km deep) discontinuity in the western part of the EE′ profile, followed by an abrupt shallowing at the easternmost edge (stations PERS-GROS).

**Fig. 8:**
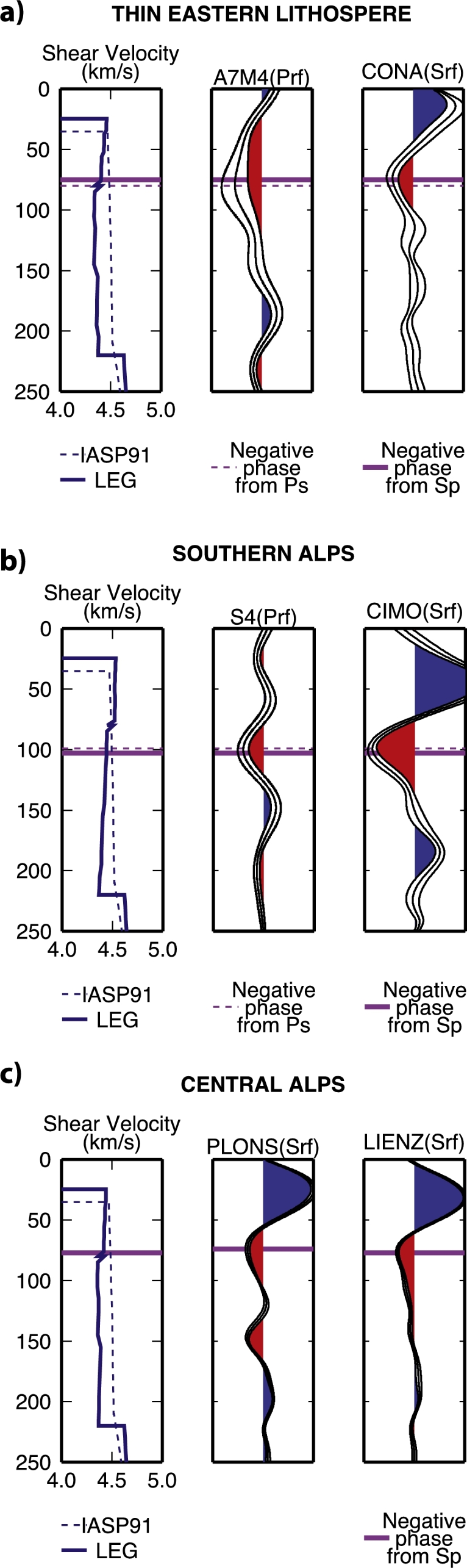
Vs at depth, PRF and SRF at six different locations in the Eastern Alps (see Fig. 1 for station locations). The negative phase is picked to best fit both Ps (dashed purple line) and Sp (purple line). It is reported on the Vs models as well. The Vs models are IASP91 used to depth migrate the PRFs and SRFs, and the Vs model from Legendre et al. (2012) for surface waves. (For interpretation of the references to color in this figure legend, the reader is referred to the web version of this article.)

We examine the single PRFs and SRFs in more detail by comparing them with the S-velocities determined from surface waves by Legendre et al. (2012). For the eastern part of the study area we compare the PRF stack from the temporary station A7M4 and SRF stack from permanent station CONA (Fig. 8). Both show a negative phase (highlighted by magenta lines) that corresponds to a velocity decrease as detected by Legendre et al. (2012), at a depth of 80 and 75 km respectively for P- and SRFs. For the Southern Alps, PRFs from “spot” 4 on profile AA′ are compared to the SRFs from permanent station CIMO (Fig. 8b). The large negative phase (associated with the discontinuity) occurs at 99 and 103 km, respectively, yet from the shear velocity model it is located at 80 km depth. In the central Alps, SRFs from stations PLONS and LIENZ (shown in Fig. 8c) the negative phase are not as broad as seen for the other stations, but the inferred depth of 75 km agrees with the lower velocity also found by Legendre et al. (2012). Although the method used in this work is sensitive to small-scale lateral changes and the LAB depth variations across the Eastern Alps are not easily identified using the surface-wave based technique employed by Legendre et al. (2012), it is useful to compare the results.

**Fig. 9:**
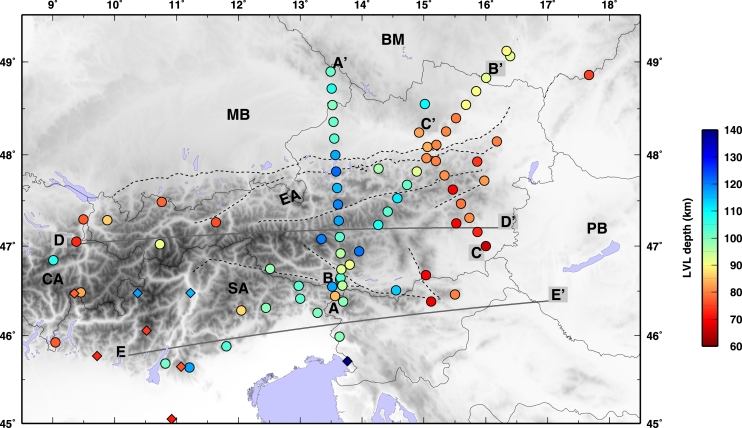
Depth of the seismic discontinuity under the Eastern Alps. The location of the profiles shown in Fig. 4, Fig. 7 are shown as reference. Circles for depths inferred from this study, diamonds for depths from Miller and Piana Agostinetti (2012).

As inferred from the PRFs computed along the AA′ profile, we image a gradual deepening of the discontinuity from the Southern Alps (∼100 km depth) towards the higher mountain crests to the north, where it deepens to about 120–130 km. Below the Molasse Basin the discontinuity depth is about 100 km (profile AA′ in Fig. 7, Fig. 9). Along profile BB′ we observe the discontinuity at depths of 100–120 km along its southern extent, then it rapidly decreases in depth (up to 80 km) coinciding with the topographic lows (Fig. 7b); it deepens again below the Bohemian Massif in the northeast. Along profile CC′ the discontinuity is approximately 80 km depth with a slight deepening towards the north. The new SRF inferred depths show a constant feature in the Southern Alps, where the depths are estimated to be about 100 km (profile EE′ in Fig. 4), yet show a shallower discontinuity below the Slovenian stations (PERS-GROS). The DD′ profile (Fig. 4) outlines the depth differences beneath the central Alps, where it shallows to about 80 km.

In summary, the discontinuity identified below Eastern Alps deepens in the central part of the study area, and rises towards the Pannonian Basin and towards the western Alps.

## 4 Discussion

Due to the employment of the two techniques we are able to infer the depth of a seismic discontinuity through the central and eastern Alpine chain. The dense distribution of temporary stations allows for the identification of lateral depth variations that occur over length scales of several tens of kilometers, which are supported by the estimated discontinuity depths obtained from data recorded at permanent stations. We are able to image the deepening of a seismic discontinuity in the axial zone of the Eastern Alps, and its rise in depth towards the east. This discontinuity corresponds to a decrease of S-velocity. S-velocity drops detected by RF analysis and encountered in the shallow part of the upper mantle (down to 100–150 km) have been interpreted as the LAB (e.g. Rychert et al., 2007, Rychert and Shearer, 2009, Eaton et al., 2009, Miller and Piana Agostinetti, 2012) or as a mid-lithospheric discontinuity (MLD) (Thybo, 2006, Yuan and Romanowicz, 2010), or both (Abt et al., 2010, Fischer et al., 2010, Miller and Eaton, 2010, Lekic and Fischer, 2014, Hopper et al., 2014). In the following we compare the depth of the Vs decrease with previously detected depth of the LAB. We compare it then to the velocity anomalies from a tomography model of the area, and finally we try to combine these observations into an interpretation of the upper mantle structures.

The lithosphere and upper mantle structure in this region has been previously imaged by continent-scale studies, where the lateral variations are smoothed over hundreds of kilometers. Such studies (i.e. Artemieva et al., 2006, Jones et al., 2010, Plomerová and Babuška, 2010, and references therein) suggest the location of the LAB is at greater depths (30–50 km deeper) compared to our results. The lithospheric structure presented in this study contains much more detail than imaged in previous investigations due to the close spacing of the seismic stations (∼20 km) and longer duration of data recorded by permanent stations, therefore allowing for improved resolution of lateral variations that might have been smeared by the large-scale interpretations.

Our results have commonality with Geissler et al. (2010) where they image a thicker lithosphere (∼120 km) between 46° to 48°N and 10° to 14°E and a surrounding thin lithosphere. A comparison with the compilation of the inferred depths to the electrical LAB by Korja (2007) also shows good agreement in the area east of 15°E, where the two datasets overlap. A rapid reduction of electrical resistivity is expected to occur in accordance with presence of a very low fraction of partial melt (Ghods and Arkani-Hamed, 2000, Tirone et al., 2009), for example from pockets of melted lithosphere. There is also correlation between our discontinuity depths and the surface heat flow from Artemieva et al. (2006), the discontinuity is shallower towards the Pannonian Basin and towards south-western Germany, where the surface heat flow is higher, and presumably generated by different geothermal gradients.

The relatively abrupt seismic wave velocity decrease (on the order of 2–9%; e.g. Rychert et al., 2007, Rychert and Shearer, 2009, and references therein) at the boundary between the lithosphere and asthenosphere has been suggested to be from a temperature effect on mechanical rock properties (e.g. Faul and Jackson, 2005, Schubert et al., 1976, Stixrude and Lithgow-Bertelloni, 2005) and often the description of the lithosphere is reduced to a thermally defined layer. However, a purely thermal model predicts only a diffuse velocity transition that is inconsistent with the sharp transitions shown in this study and in many others (e.g. Rychert et al., 2007, Eaton et al., 2009, Fischer et al., 2010, Miller and Eaton, 2010, Levander and Miller, 2012).

The seismic wave conversion that we detect with receiver functions in the upper mantle may be due to the presence of non-thermal variations in the lithosphere (caused by fluids, anisotropy, composition or grain size differences).

**Fig. 10:**
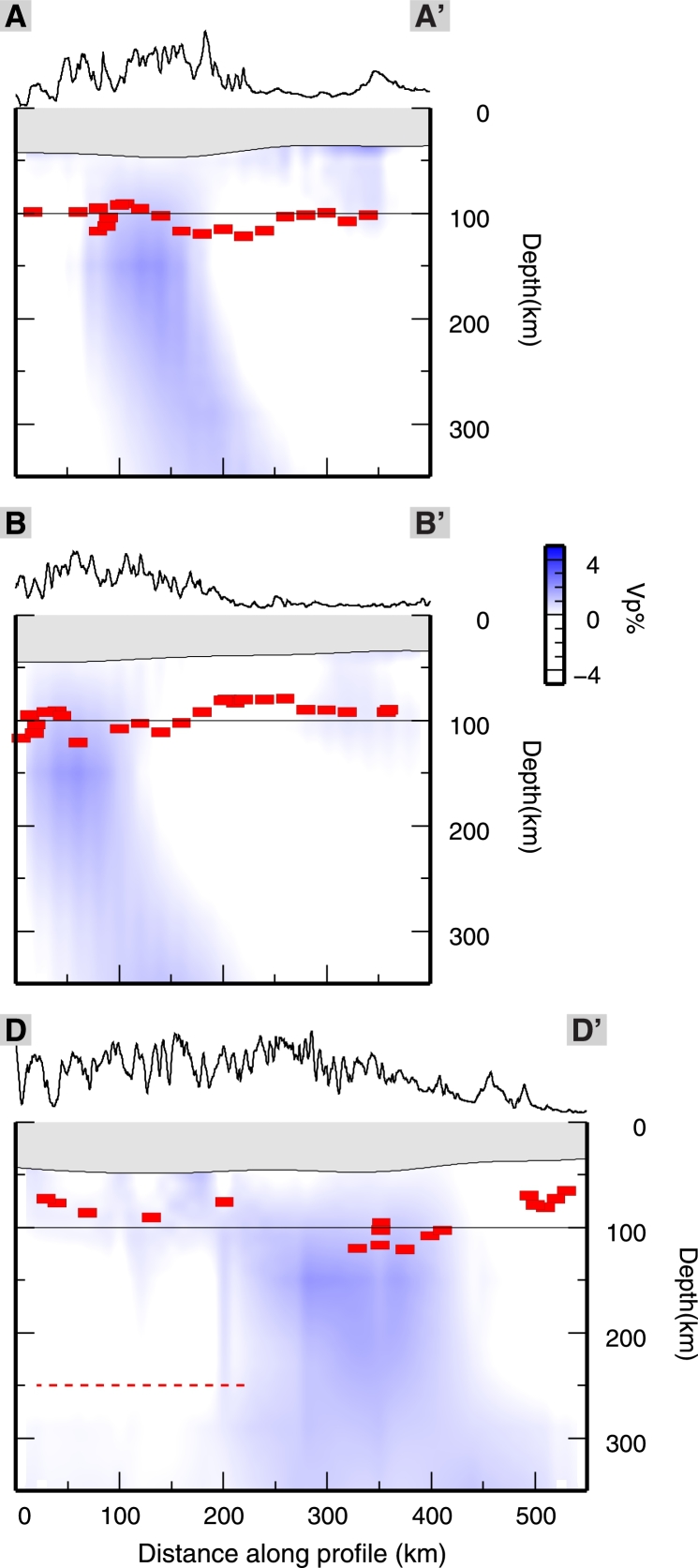
Spatial relation between tomography anomalies (Koulakov et al., 2009) and discontinuity depth along the three profiles AA′, BB′, DD′. On profile DD′, depth of the discontinuity at a distance of ±30 km are shown, on profile AA′ and BB′ depths obtained at a distance of ±20 km are included. Gray area on profiles shows estimated crustal thickness from Grad et al. (2009).

The detected discontinuity is deeper in areas where the fast velocities associated with the downgoing slab are detected by seismic tomography (i.e. Lippitsch et al., 2003, Koulakov et al., 2009, Mitterbauer et al., 2011). The influence of the subduction is reflected in the presence of thicker lithosphere, along the axial zone of the Alpine chain, where crustal and mantle material is involved in the construction of the mountain chain and its roots after the indentation process. Receiver functions allow detecting the presence of discontinuities that might be invisible to other geophysical investigations, especially seismic tomography. The occurrence of high velocity anomalies is clearly detected by Lippitsch et al. (2003), Koulakov et al. (2009), Mitterbauer et al. (2011), Karousová et al. (2012), but the tomographic models show some differences at depth. However in seismic tomography it is nearly impossible to verify the presence of discontinuities or small-scale variations, which are instead the basis of the RF technique; a joint interpretation can provide further constraints on mantle architecture. The comparison with the high velocity anomalies from the tomographic models and the retrieved depths, suggests that the discontinuity resides at the top or partially cuts the positive anomaly at shallow depth (at 100 to 130 km depth in Fig. 10, AA′ and DD′ profiles). A velocity decrease atop the subducting body has been observed in cases of oceanic subduction (e.g. Abers, 2005, Rondenay et al., 2008), nevertheless the occurrence of a velocity decrease due to subduction does not explain its occurrence outside of the subducting body. Recently Karato (2012) argued for the existence of a substantial velocity drop due to grain boundary sliding, and encountered at temperature of ∼1300 K (corresponding to depths of ∼100–150 km in the typical old continental upper mantle; Artemieva, 2009). This model suggests the presence of a ∼5% or larger velocity drop described as MLD while the continental LAB (as viscosity contrast) would be deeper lacking of a strong velocity reduction. This argument fits our observations considering the deepening of the interpreted negative velocity boundary within the occurrence of the tomographic high velocity bodies, as due to temperature cooling. With the actual knowledge we are far away from stating whether the discontinuity actually cuts the slab, and whether there is a slab detachment.

Either of the discussed interpretations may fit to our results. The discontinuity might represent the LAB or correspond to the discontinuity illustrated by Karato (2012) which is deeper within the slab. To the west of 12°E, two discontinuities have been recognized, both due to velocity decrease (Fig. 10, profile DD′); the shallow is at 80–90 km depth conceivably related to the MLD; and the deeper (∼250 km) fits to a low velocity anomaly detected by tomography, possibly due to the LAB. Between 12° and 15°E the influence of the slab increases the depth of the discontinuity, suggesting the influence of colder temperature on the rheological behavior of the mantle at these depths (as seen in Karato, 2012). East of 15°E the velocity decrease and LAB coincide. At these longitudes, the lithosphere is thinner, and the eastward extrusion of the Eastern Alps (Ratschbacher et al., 1991b, Ratschbacher et al., 1991a) can be recalled to support this observation. The extrusion as acting on the entire lithospheric block may have caused the entire lithosphere to thin, or an already thinner lithosphere might have accommodated the extrusion process.

## 5 Conclusions

In the Eastern Alps, teleseismic events recorded at a dense network of permanent and temporary seismic stations have been exploited to create receiver functions. Both PRFs and SRFs show a coherent phase that gives consistent results for the lithospheric structure. The converted phase is due to a sharp velocity decrease that occurs at variable depth across the study region. Our discontinuity depth estimates and those of the lithosphere–asthenosphere boundary (LAB) detected by previous studies (Artemieva et al., 2006, Korja, 2007, Geissler et al., 2010) are comparable, but our results show topographic variation at smaller scale of this surface.

The discontinuity is deepest beneath the central Eastern Alps (120–130 km), then shallows towards the Molasse Basin and towards the Southern Alps (∼100 km depth), then its depth decreases to the west to approximately 80–90 km. Due to the occurrence of high velocity anomalies from tomographic studies in the area, we interpret this as a lithospheric low velocity zone. This interpretation is reinforced by the occurrence of a second discontinuity at greater depths (∼250 km) that might represent the LAB (west of 12°E). We interpret that discontinuity as the lithosphere/asthenosphere boundary (LAB) for the easternmost part of the study area, east of 15° longitude, where it is 70–80 km deep, in this area lithospheric thinning towards the Pannonian Basin supports lateral extrusion.

## Appendix A Supplementary material

MMC 1 Table T1: Depth of the discontinuity and depth uncertainty retrieved at each station. Fig. S1: Events distribution for station ACOM and “spot 6” from profile BB′. Fig. S2: Average amplitude spectra of the vertical component recorded at station A306 and A109. Fig. S3: Synthetic PRFs calculated according to the velocity models in Table T2. Table T2: Velocity models used to compute synthetic PRFs.
