# Supplementary material for: Insights on the upper mantle beneath the Eastern Alps
Source: Earth Planet Sci Lett. 2014 Oct 1;403:199–209. doi: 10.1016/j.epsl.2014.06.051 (PMC4375711; doi:10.1016/j.epsl.2014.06.051)
Supplement: MMC 1 — Table T1: Depth of the discontinuity and depth uncertainty retrieved at each station. Fig. S1: Events distribution for station ACOM and “spot 6” from profile BB′. Fig. S2: Average amplitude spectra of the vertical component recorded at station A306 and A109. Fig. S3: Synthetic PRFs calculated according to the velocity models in Table T2. Table T2: Velocity models used to compute synthetic PRFs. [file mmc1.pdf]

SUPPLEMENTARY ONLINE MATERIAL

Table S1: Station name, coordinates, depth of the discontinuity and depth uncertainty.

STATION	LON	LAT	LAB DEPTH (km)	FW95M	
				Min (km)	Max (km)
JAVC	17.670	48.859	75	70	81
KRUC	16.395	49.061	92	86	99
ACOM	13.514	46.548	117	111	123
AGOR	12.047	46.282	87	82	94
BALD	10.818	45.683	101	95	108
CGRP	11.804	45.880	104	96	110
CIMO	12.444	46.311	99	93	105
FUSE	13.001	46.414	103	97	110
MARN	11.209	45.637	117	111	122
SABO	13.633	45.987	99	93	105
VINO	13.281	46.256	100	93	109
ZOU2	12.972	46.558	103	96	113
ABTA	12.512	46.747	98	94	105
ARSA	15.523	47.250	70	66	77
CONA	15.861	47.928	74	69	80
DAVA	9.880	47.286	86	82	90
FETA	10.729	47.021	91	87	98
KBA	13.344	47.078	120	115	125
MOA	14.265	47.849	97	89	108
MYKA	13.641	46.629	112	105	118
OBKA	14.548	46.509	114	110	121
RETA	10.762	47.487	78	75	85
SOKA	15.033	46.678	70	66	83
WTTA	11.636	47.263	76	72	81
GROS	15.502	46.461	80	69	88
PERS	15.117	46.381	69	64	76
LIENZ	9.494	47.296	77	74	79
LLS	9.009	46.843	107	103	111
PLONS	9.381	47.049	73	69	80
VDL	9.451	46.485	83	79	90
MUGIO	9.042	45.922	77	74	81
**PROFILE AA'**					
S1	13.735	45.843	97	91	103
S2	13.721	46.022	124	104	141
S3	13.708	46.202	103	100	109
S4	13.694	46.382	100	95	105
S5	13.680	46.561	95	87	102
S6	13.666	46.741	92	85	98
S7	13.652	46.920	96	90	102
S8	13.638	47.100	102	95	108
S9	13.638	47.100	116	102	130

S10	13.610	47.459	119	103	135
S11	13.595	47.639	115	102	126
S12	13.581	47.818	121	103	138
S13	13.566	47.998	116	103	128
S14	13.551	48.178	103	96	110
S15	13.537	48.357	101	96	107
S16	13.522	48.537	100	93	105
S17	13.507	48.716	105	101	109
**PROFILE BB'**					
S2	13.650	46.647	102	97	108
S3	13.801	46.794	92	87	99
S4	13.953	46.941	121	104	136
S6	14.260	47.234	108	102	114
S7	14.4141	47.380	103	97	108
S8	14.569	47.526	111	102	120
S9	14.726	47.672	102	94	111
S10	14.883	47.818	92	84	100
S11	15.041	47.963	79	73	85
S12	15.200	48.108	81	75	86
S13	15.359	48.253	81	75	86
S14	15.520	48.398	79	74	85
S15	15.681	48.542	91	86	95
S17	16.007	48.831	89	81	96
**PROFILE CC'**					
S1	16.000	47.000	66	61	70
S2	15.868	47.156	73	68	77
S3	15.735	47.311	78	73	83
S4	15.601	47.467	78	76	80
S5	15.466	47.622	72	64	79
S6	15.331	47.777	81	74	89
S7	15.195	47.932	80	74	89
S8	15.058	48.087	84	78	90
S9	14.920	48.241	82	77	87

Uncertainty of depth estimates

The uncertainty on the discontinuity depths depends on the data quality, the technique resolving power, and on the velocity model used for the depth migration. Here, we estimate the uncertainty given by each of these three factors. Depth uncertainties due to the data quality, as reflected in the variation among the receiver functions, were estimated for the stacked RF by bootstrapping as in Abt et al. (2010) and Miller and Piana Agostinetti (2012). For both PRFs and SRFs at each station, the initial waveform gather is replaced by an equal number of randomly selected waveforms, and the stacked receiver functions were recalculated. This process was repeated 10 times for the SRF and 20 times for the PRF and the standard deviation of the resulting set of receiver

functions was calculated. We consider the well-resolved portions of the receiver functions to be those still for positive amplitudes below the rf-σ and negative amplitudes above the rf+σ.

Concerning the resolving power of the technique, specifically the finite frequencies of the observations, the uncertainties on depth estimates are calculated at the 95% of the maximum of the negative pulse. The Full-Width-95%-Maximum (FW95M) gives the width across the negative pulse when it drops to 95% of its peak, or minimum, value. In Table S1 we show minimum and maximum depths retrieved by the FW95M, whose values describe the peak sharpness and the LVD depth range. The broadest pulse is found at station MOA and GROS, where the FW95M gives a 19 km depth range. The sharpest pulse is found at station S4 in Profile CC' where the FW95M gives 4 km of depth uncertainty. On average the SRFs have ±6 km depth uncertainty, while the PRFs have a ±8 km depth uncertainty.

The effects of errors in the velocity model on the depth estimate are smaller with respect to the resolving power of the technique. Considering an average discontinuity depth of 100 km, and a 5% error in the velocity model employed for migration, we obtain about 5 km difference in depth estimate, which is smaller than the depth uncertainty estimated for the negative signal in the P- and SRFs. This consideration on the uncertainties added by the employment of an unsuitable velocity model, has been explored in previous work (e.g. Zhai and Levander, 2011; Levander and Miller, 2012).

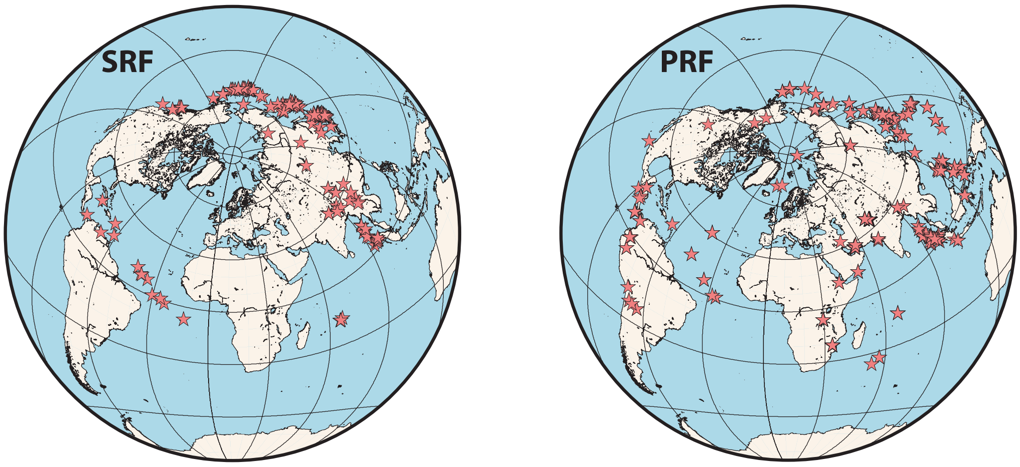

FIGURE S1. Events distribution for example station ACOM (see Figure 1 for station location) for SRFs (154 events), and "spot 6" (see Figure 1 for station location) from profile BB' in Figure 1 for PRFs (371 events).

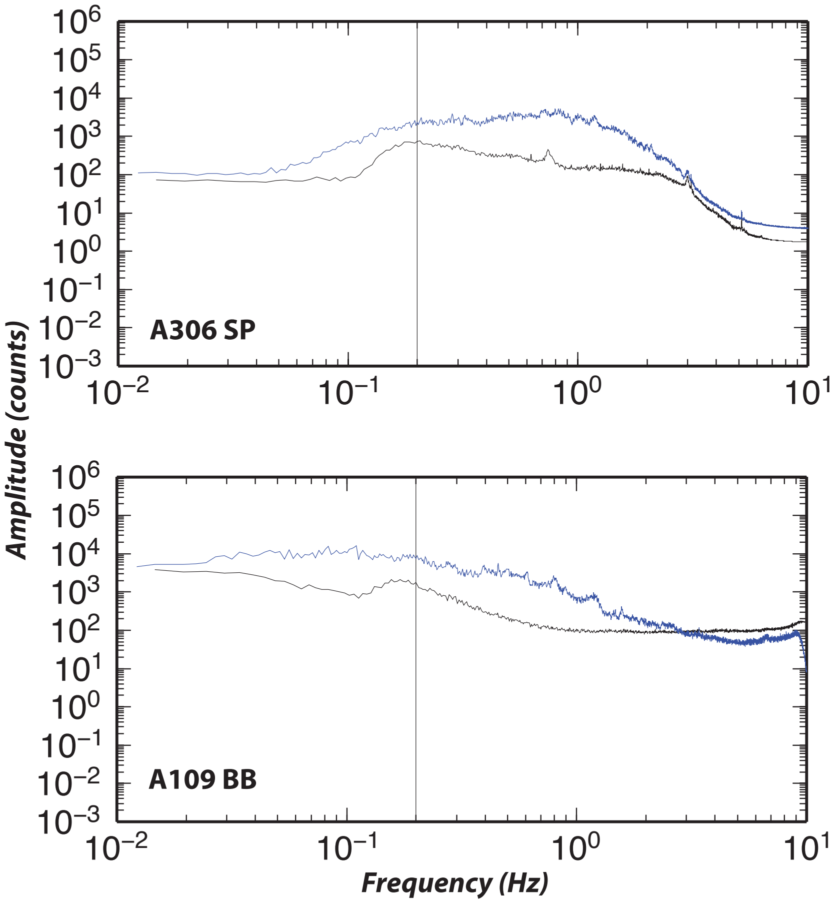

FIGURE S2. Average amplitude spectra of the vertical component of earthquakes (blue) and pre-event noise (black) recorded at stations A306 (short period) and A109 (broadband).

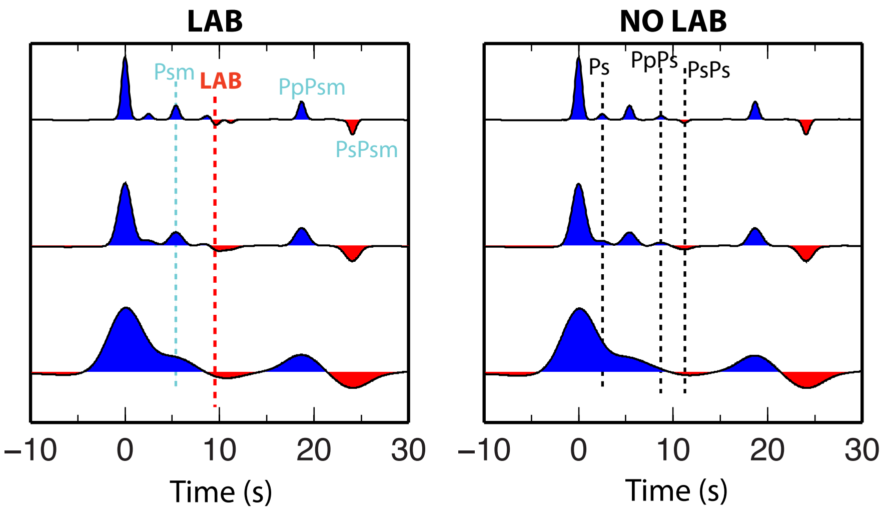

FIGURE S3. Synthetic PRF calculated according to the velocity models in Table T2. Wiggles are obtained according to different cut-off frequencies (1, 0.5 and 0.2 Hz from top to bottom). In figure the Ps phase due to the interface at 20 km, and its multiples, are named and marked on the right panel, while LAB and Moho phases are named and marked on the left panel. 6% velocity decrease at 80 km depth gives quite a small conversion but it's still visible.

Table T2. Velocity models used to compute synthetic PRF in Figure S3.

Thickness (km)	Density (g/cm^3)	Vs (km/s)	Vp/Vs
	**LAB**		
20	2.8	3.5	1.73
25	2.8	3.8	1.73
40	3.3	4.48	1.81
--	3.3	4.2	1.81
	**NO LAB**		
20	2.8	3.5	1.73
25	2.8	3.8	1.73
40	3.3	4.48	1.81
--	3.3	4.48	1.81

References

Abt, D. L., K. M. Fischer, S. W. French, H. A. Ford, H. Yuan, *and* B. Romanowicz *(*2010*),* North American lithospheric discontinuity structure imaged by *Ps* and *Sp* receiver functions, J. Geophys. Res., 115, B09301, doi:10.1029/2009JB006914.

Levander, A. and Miller, M.S., (2012), Evolutionary aspects of lithosphere discontinuity structure in the western U.S., Geochemistry, Geophysics, Geosystems, 13, doi:10.1029/2012GC004138.

Miller, M. S. Piana Agostinetti N. 2012. Insights into the evolution of the Italian lithospheric structure from S receiver function analysis. Earth and Planetary Science Letters, Volumes 345–348, September 2012, Pages 49-59

Zhai, Y., Levander, A., 2011. Receiver function imaging in strongly laterally heterogeneous crust: synthetic modeling of BOLIVAR data. Earthq. Sci. 24, 45–54.
